# Characterization of left and right atrial function in healthy volunteers by cardiovascular magnetic resonance

**DOI:** 10.1186/s12968-016-0284-8

**Published:** 2016-10-10

**Authors:** Alicia M. Maceira, Juan Cosin-Sales, Sanjay K. Prasad, Dudley J. Pennell

**Affiliations:** 1 Cardiovascular Imaging Unit, ERESA Medical Center, MR Unit, Hospital Arnau de Vilanova, Valencia, RIC Spain; 2 Department of Cardiology, Hospital Arnau de Vilanova, Valencia, Spain; 3 Cardiovascular Magnetic Resonance Unit, Royal Brompton Hospital, London, UK; 4 NIHR Cardiovascular Biomedical Research Unit, Royal Brompton and Harefield NHS Foundation Trust and Imperial College, London, UK; 5 Department of Medicine, Health Sciences School, CEU Cardenal Herrera University, Valencia, Spain

**Keywords:** Cardiovascular magnetic resonance, Heart, Left atrium, Right atrium, Reservoir, Conduit, Pump function, Reference values

## Abstract

**Background:**

Left and right atrial function show a different pattern in advanced age in order to maintain adequate ventricular filling. It has been shown that left atrial (LA) function has a prognostic value in a number of heart conditions. Cardiovascular magnetic resonance (CMR) provides high quality images of the left and right atria using high temporal resolution steady state free precession (SSFP) cine sequences. We used SSFP cines to characterize atrial function in healthy, normotensive, volunteers.

**Methods:**

We measured maximum, preatrial contraction and minimum left and right atrial volumes in 120 healthy subjects after careful exclusion of cardiovascular abnormality (60 men, 60 women; 20 subjects per age decile from 20 to 80 years). Data were generated from 3-dimensional modeling, including tracking of the atrioventricular ring motion and time-volume curves analysis. With those measurements, all the usual parameters for left and right atrial function were calculated.

**Results:**

Gender had significant influence on some parameters of left and right atrial conduit and booster pump function. Age significantly influenced the majority of parameters of both left and right atrial function, with typically lower reservoir and conduit functions and higher booster pump function, both in males and females belonging to older age groups. CMR normal ranges were modelled for clinical use with normalization, where appropriate, for body surface area and gender, displaying parameters with respect to age.

**Conclusions:**

CMR normal reference ranges for components of left and right atrial function are provided for males and females for a wide age range.

## Background

The cardiac atria are highly dynamic chambers with neurohormonal connections and a pivotal role in the modulation of left ventricular filling. It has been shown that changes in left atrial (LA) function have a prognostic value in conditions such as ischemic heart disease [1, 2], heart failure [3], non-ischemic cardiomyopathies [4, 5], aortic stenosis [6], hypertension [7] and especially in atrial fibrillation [8–10]. Currently, with the implementation of imaging techniques that are accurate for the measurement of atrial function, this is being increasingly carried out in daily clinical practice. Atrial function has been conventionally divided into three components: first, as a reservoir, the atria store venous blood during ventricular contraction and isovolumetric relaxation; second, as a conduit, blood flows passively into the ventricles; third, as a pump, the atria contract during the final phase of diastole to boost ventricular filling. Since atrial function varies with age and other influences, it is important to obtain the normal reference range of atrial function parameters for clinical use.

Most studies on atrial function have been published with echocardiography, mainly with 2D techniques, but more recently also with 3D techniques that allow more detailed assessment of atrial function. Also, atrial strain and strain rate analysis using either tissue Doppler imaging or two-dimensional speckle tracking echocardiography have proved to be feasible and reproducible to evaluate LA mechanics [11–13]. But there are not many reports in the literature with respect to analysis of left, and right, atrial function. Cardiovascular magnetic resonance (CMR) is the gold standard technique for measurement of atrial and ventricular dimensions, for which we have previously published reference ranges for all cardiac chambers in adults using the Steady State Free Precession (SSFP) cine sequences [14–17]. Therefore, the aim of this study was to establish SSFP-based reference values for LA and right atrial (RA) function parameters normalized for independent influences such as age, gender and body surface area when required.

## Methods

### Healthy volunteers

Between 2002 and 2003, 120 subjects, with 10 men and 10 women in each of 6 age deciles from 20 to 80 years, were studied with CMR. The baseline characteristics of these healthy subjects have been previously published [14]. Briefly, all subjects were normotensive, asymptomatic, with no known risk factors or history of cardiac disease, and normal physical examination and electrocardiogram. Serum samples were also obtained at the time of that CMR scan, stored at −80 °C for up to 6 months and used for determination of serum levels of BNP (brain natriuretic peptide). By calculation of their coronary artery disease risk over 10 years [18] and quantification of BNP levels, all the volunteers were considered to have a normal cardiovascular system.

### CMR

CMR was performed with 1.5 T scanners (Siemens Sonata, Erlangen, Germany) using front and back surface coils and retrospective ECG triggering for capture of the entire cardiac cycle including diastole. All CMR scans were acquired by the same operator. SSFP end-expiratory breath-hold cines were acquired in the 2, 4 and 3 chamber views, with subsequent contiguous short-axis cines from the atrioventricular (AV) ring to the base of the atria with slice thickness of 5 mm and no gap between slices. The temporal resolution was 21 ± 1 ms. Sequence parameters included repetition time/echo time of 3.2/ 1.6 ms, in-plane pixel size of 2.1 x 1.3 mm, flip angle 60°, and acquisition time of typically 18 heartbeats. For ventricular volume acquisition the same parameters were used except slice thickness and interslice gap of 7 mm and 3 mm, respectively.

### CMR analysis

**Fig. 1:**
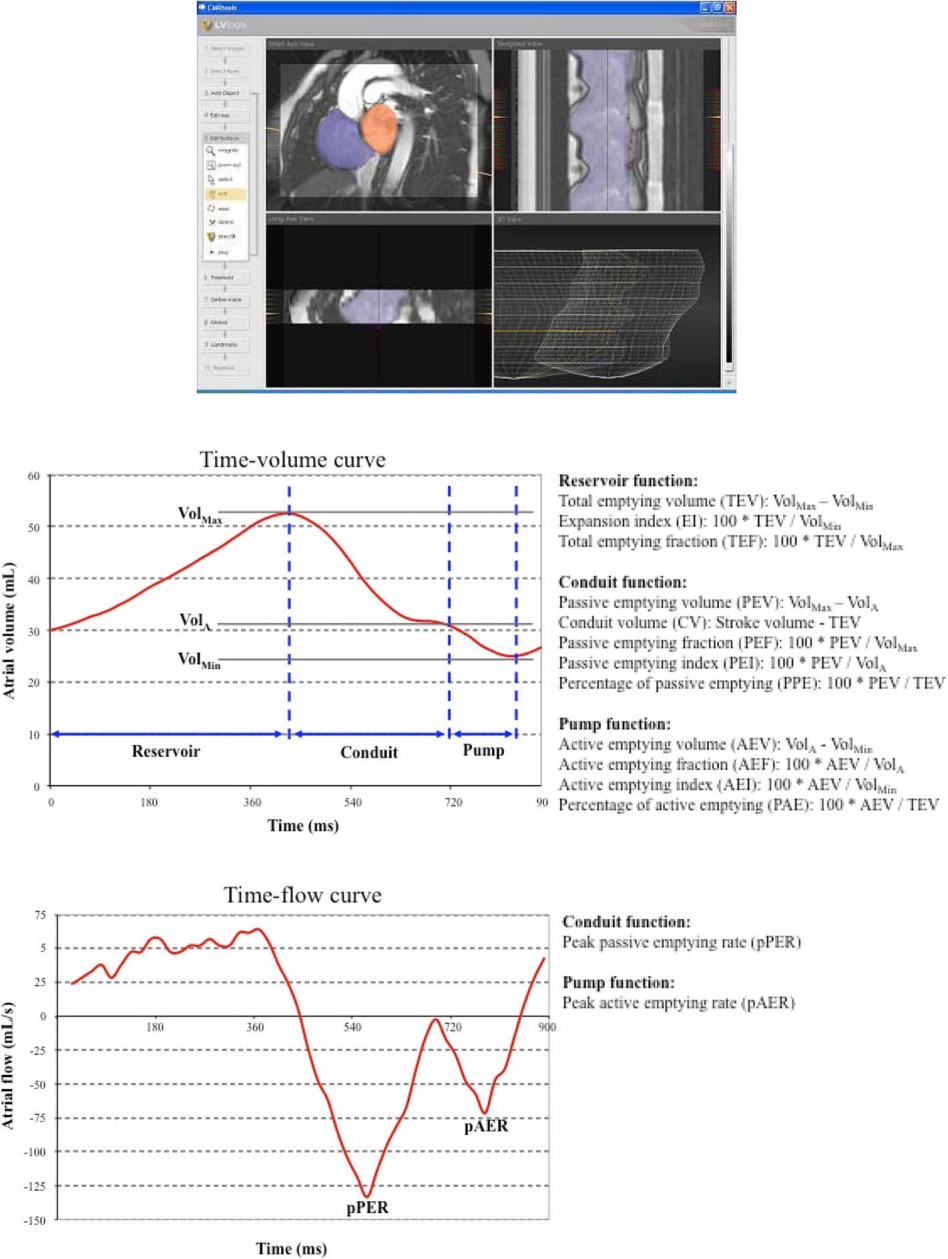
Graphs showing software analysis of atrial volumes (*top*), atrial time-volume curve (*middle, left*) and time-flow curve (*bottom, left*) from which atrial function parameters (*middle and bottom, right*) are derived

Analysis was performed with a personal computer and semi-automated software (CMRTools, Cardiovascular Imaging Solutions, London, UK). In all the healthy volunteers maximum atrial volumes as well as ventricular volumes were measured and have been previously reported [16, 17]. Atrial volume analysis included delineation of the atrial endocardial borders, in all planes in all cardiac phases, and calculation of the systolic descent and twist of the AV valves from tracking of the valve motion on the long axis cines, which was used to correct for increase in atrial volume due to AV ring descent. In the analysis we included the atrial appendages and excluded the pulmonary and cava veins [16]. With this information a time-volume curve was produced, in which phasic volumes (maximum, preatrial contraction, minimum) were measured, and a time-flow curve was also derived (Fig. 1).

The following parameters of atrial function were calculated for the reservoir, conduit and pump components [19], which are shown in Fig. 1:

For reservoir function: total emptying volume, also called reservoir volume or cyclic volume change (TEV), total emptying—or ejection—fraction (TEF) and expansion index, also called total emptying index (EI).

For conduit function: passive emptying volume, which represents early diastolic filling (PEV), conduit volume, which is the amount of blood that transits the atria into the ventricles while the atrioventricular valves are open (CV), passive emptying—or ejection—fraction (PEF), passive emptying index (PEI), percentage of passive emptying (PPE) and peak passive emptying rate (pPER).

For booster pump function: active emptying volume, which represents late diastolic filling due to atrial contraction (AEV), active emptying—or ejection—fraction (AEF), active emptying index (AEI), percentage of active emptying (PAE) and peak active emptying rate (pAER). Finally, the ratio of active to passive emptying volumes was calculated (AEV/PEV).

### Statistical analysis

All the CMR derived parameters were found to satisfy a normal distribution using the Kolmogorov-Smirnov test and summary data for these variables are therefore presented as mean ± SD. BNP was normally distributed after log-transformation. Simple linear regression was used to analyse variations in atrial function parameters and plasmatic markers due to age and gender, to model the data and to construct reference ranges as mean and 95 % confidence intervals, which were generated by adding or subtracting 1.96 * standard deviation to the mean. Two-way ANOVA was used to analyse variations in parameters due to age and gender. *P* values <0.05 were considered significant. Differences in LA and RA function parameters according to age and gender as well as correlations with these variables, were analysed.

## Results

The baseline characteristics of the subjects included have been reported previously, as stated above [14]. Briefly, the volunteers were 10 males and 10 females for each age decile from 20 to 80 years. Average values for age, height, weight and body mass index were 49 ± 17 years, 1.71 ± 0.9 m, 72 ± 13 kg, 24 ± 4 kg/m^2^. Mean heart rate was 66 ± 10 bpm, mean systolic and diastolic blood pressures were 124 ± 12 mmHg, 73 ± 7 mmHg.

### Parameters of LA function

**Fig. 2:**
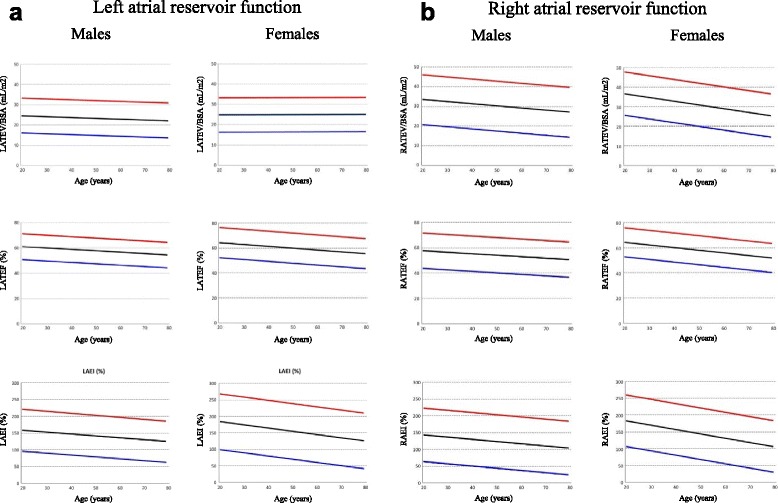
Graphs showing left (**a**) and right (**b**) atrial reservoir function parameters. For each parameter mean (*black lines*), upper (*red lines*) and lower (*blue lines*) 95 % confidence intervals are depicted

**Fig. 3:**
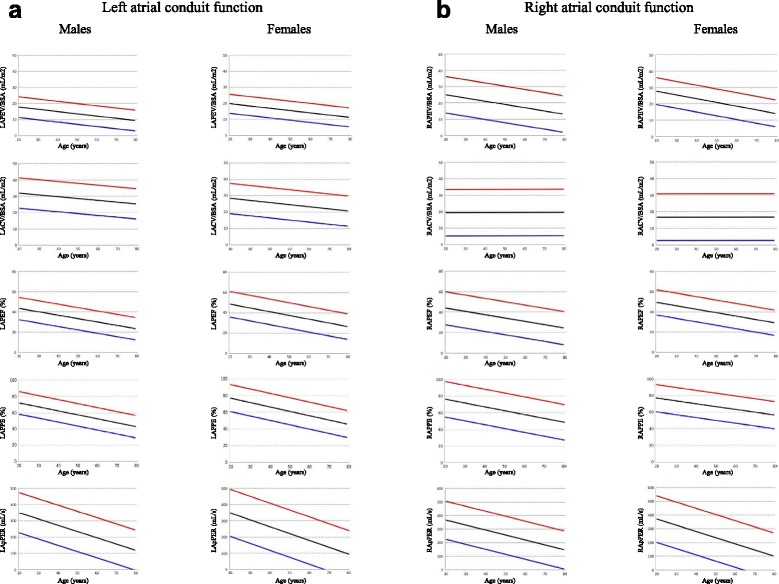
Graphs showing left (**a**) and right (**b**) atrial conduit function parameters. For each parameter mean (*black lines*), upper (*red lines*) and lower (*blue lines*) 95 % confidence intervals are depicted

**Fig. 4:**
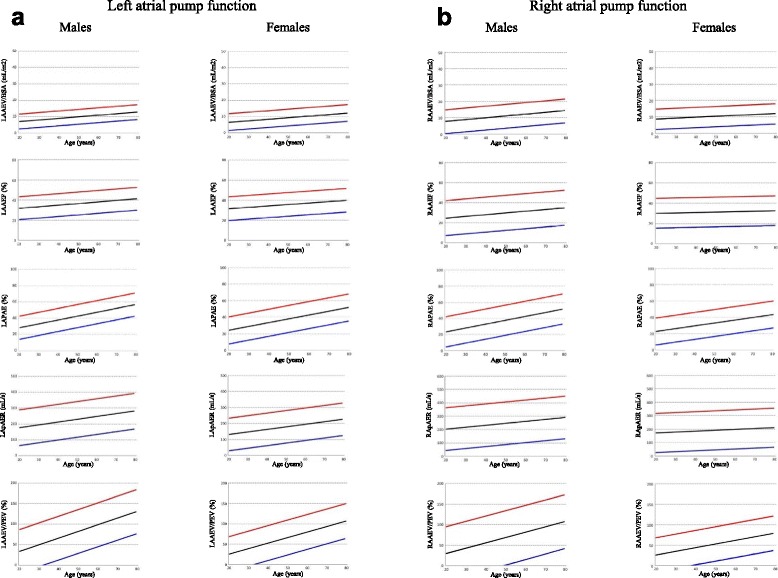
Graphs showing left (**a**) and right (**b**) atrial pump function parameters. For each parameter mean (*black lines*), upper (*red lines*) and lower (*blue lines*) 95 % confidence intervals are depicted

The results summarized for the entire study group, and male and female groups, without age breakdown, are shown in Table 1, with sub-division into absolute and body surface area (BSA) normalized values, for application to studies of unsorted subjects. For those variables with significant differences with age, results across age deciles are shown for the whole group (Table 2) and for females and males (Tables 3 and 4). Figs. 2a, 3a and 4a show the main parameters plotted against age. Variables significantly affected by BSA are presented normalized by this variable.

**Table 1:** Left atrial function reference parameters summary data for all ages (mean, 95 % confidence interval)

	All	Males	Females
LATEV [cmL] SD 8.9	44 (27, 62)	46 (28, 64)	42 (27, 58)
LATEV/BSA [mL/m^2^] SD 4.4	24 (15, 33)	24 (15, 33)	25 (17, 33)
LATEF [%] SD 5.8 *	59 (47, 70)	58 (47, 68)	60 (48, 72)
LAEI [%] SD 37.8 * **	148 (74, 122)	141 (79, 203)	156 (71, 240)
LAPEV [mL] SD 6.4 * **	27 (14, 39)	27 (14, 39)	26 (14, 38)
LAPEV/BSA [mL/ m^2^] SD 3.1	14 (8, 20)	14 (7, 20)	15 (9, 21)
LACV [mL] SD 9.6 * **	49 (30, 68)	56 (36, 76)	42 (25, 59)
LACV/BSA [mL/m^2^] SD 4.7	27 (17, 36)	29 (18, 38)	24 (15, 34)
LAPEF [%] SD 6.0 * **	35 (24, 47)	33 (22, 44)	37 (25, 50)
LAPEI [%] SD 16.5 * **	60 (28, 93)	52 (26, 78)	63 (25, 101)
LAPPE [%] SD 7.7 * **	60 (45, 75)	57 (43, 71)	61 (45, 78)
LApPER [mL/s] SD 68.4 *	228 (94, 362)	234 (111, 357)	221 (76, 366)
LApPER/BSA [mL/s/m^2^] SD 35.5	179 (109, 248)	120 (56, 184)	129 (55, 203)
LAAEV [mL] SD 4.7 *	18 (9, 27)	19 (11, 28)	16 (6, 26)
LAAEV/BSA [mL/m^2^] SD 2.4	10 (5, 14)	10 (5, 14)	9 (4, 14)
LAAEF [%] SD 6.8 *	36 (23, 49)	37 (25, 48)	36 (24, 48)
LAAEI [%] SD 15.9 *	58 (27, 88)	59 (32, 86)	57 (25, 89)
LAPAE [%] SD 7.7 * **	40 (25, 55)	43 (29, 57)	38 (22, 54)
LApAER [mL/s] SD 54.5 *	204 (97, 311)	229 (117, 342)	179 (77, 280)
LApAER/BSA [mL/s/m2] SD 28.4	110 (54, 166)	117 (60, 174)	104 (49, 158)
LAAEV/LAPEV [%] SD 24.7 *	74 (26, 123)	82 (29, 135)	66 (23, 109)

*LA* left atrium, *TEV* total emptying volume, *BSA* body surface area, *EI* expansion index, *TEF* total emptying fraction, *PEV* passive emptying volume, *CV* conduit volume, *PEF* passive emptying fraction, *PEI* passive emptying index, *pPER* peak passive emptying rate, *PPE* percentage of passive emptying, *AEV* active emptying volume, *AEF* active emptying fraction, *AEI* active emptying index, *pAER* peak active emptying rate, *PAE* percentage of active emptying, *SD* standard deviation for the whole group

* Significant differences (*p <* 0.05) among age groups on multivariate analysis

** Significant differences (*p <* 0.05) between males and females on multivariate analysis

**Table 2:** Left atrial function parameters significantly influenced by age in the whole group (mean, 95 % confidence interval)

	20–29 years	30–39 years	40–49 years	50–59 years	60–69 years	70–79 years
LATEF [%] SD 5.8	62 (51, 74)	61 (50, 72)	60 (48, 71)	58 (47, 70)	57 (45, 68)	55 (44, 67)
LAEI [%] SD 37.7	170 (96, 244)	162 (88, 236)	153 (79, 227)	145 (71, 219)	137 (63, 211)	128 (54, 203)
LAPEV [mL] SD 6.3	33 (20, 45)	30 (18, 43)	28 (15, 40)	26 (13, 38)	23 (11, 36)	21 (8, 33)
LAPEV/BSA [mL/ m^2^] SD 3.1	18 (12, 24)	17 (10, 23)	15 (9, 21)	14 (8, 20)	12 (6, 18)	11 (5, 17)
LACV [mL] SD 9.6	54 (35, 73)	52 (33, 71)	50 (31, 69)	48 (29, 67)	46 (27, 65)	44 (25, 63)
LACV/BSA [mL/m^2^] SD 4.7	30 (20, 39)	28 (19, 38)	27 (18, 36)	26 (17, 35)	25 (16, 34)	24 (14, 33)
LAPEF [%] SD 6.0	44 (33, 56)	41 (29, 53)	37 (26, 49)	34 (22, 46)	30 (19, 42)	27 (15, 39)
LAPEI [%] SD 16.5	83 (50, 115)	74 (42, 107)	65 (33, 98)	57 (24, 89)	48 (15, 80)	39 (7, 72)
LAPPE [%] SD 7.7	72 (57, 87)	67 (52, 82)	62 (47, 78)	58 (43, 73)	53 (38, 78)	48 (33, 63)
LApPER [mL/s] SD 68.4	333 (199, 467)	292 (158, 426)	252 (118, 386)	211 (77, 346)	171 (37, 305)	131 (−3, 265)
LApPER/BSA [mL/s/m^2^] SD 35.5	183 (114, 253)	161 (91, 230)	138 (68, 207)	115 (45, 185)	92 (23, 162)	69 (0, 139)
LAAEV [mL] SD 4.7	13 (4, 22)	15 (6, 24)	17 (7, 26)	19 (9, 28)	20 (11, 30)	22 (13, 31)
LAAEV/BSA [mL/m^2^] SD 2.5	7 (2, 12)	8 (3, 13)	9 (4, 14)	10 (5, 15)	11 (6, 16)	12 (7, 17)
LAAEF [%] SD 6.8	32 (19, 46)	34 (20, 47)	35 (22, 49)	37 (23, 50)	38 (25, 51)	39 (26, 53)
LAAEI [%] SD 15.9	53 (22, 84)	57 (25, 88)	60 (29, 91)	64 (32, 95)	67 (36, 98)	61 (39, 102)
LAPAE [%] SD 7.7	28 (13, 43)	33 (18, 48)	38 (22, 53)	42 (27, 57)	47 (32, 62)	52 (37, 67)
LApAER [mL/s] SD 54.4	161 (54, 268)	178 (71, 284)	194 (87, 301)	211 (104, 318)	227 (121, 334)	244 (137, 351)
LApAER/BSA [mL/s/m2] SD 28.4	88 (33, 144)	97 (41, 152)	105 (49, 161)	113 (58, 169)	122 (66, 177)	130 (74, 186)
LAAEV/LAPEV [%] SD 24.7	35 (−13, 84)	50 (2, 99)	65 (17, 114)	80 (32, 129)	95 (47, 144)	110 (62, 159)

*LA* left atrium, *BSA* body surface area, *EI* expansion index, *TEF* total emptying fraction, *PEV* passive emptying volume, *CV* conduit volume, *PEF* passive emptying fraction, *PEI* passive emptying index, *pPER* peak passive emptying rate, *PPE* percentage of passive emptying, *AEV* active emptying volume, *AEF* active emptying fraction, *AEI* active emptying index, *pAER* peak active emptying rate, *PAE* percentage of active emptying

**Table 3:** Left atrial function parameters significantly influenced by age in females (mean, 95 % confidence interval)

	20–29 years	30–39 years	40–49 years	50–59 years	60–69 years	70–79 years
LATEF [%] SD 6.1	64 (52, 76)	62 (50, 74)	61 (49, 73)	59 (47, 71)	58 (46, 70)	56 (44, 68)
LAEI [%] SD 42.8	184 (100, 268)	173 (89, 257)	162 (78, 246)	151 (67, 235)	140 (56, 224)	129 (45, 213)
LAPEV [mL] SD 6.2	32 (22, 44)	30 (18, 42)	28 (16, 40)	25 (13, 38)	23 (11, 35)	21 (9, 33)
LAPEV/BSA [mL/ m^2^] SD 3.1	19 (13, 25)	18 (12, 23)	16 (10, 22)	15 (9, 21)	13 (7, 19)	12 (6, 18)
LACV [mL] SD 8.9	47 (30, 65)	45 (28, 63)	43 (26, 61)	41 (24, 59)	39 (22, 57)	37 (20, 55)
LACV/BSA [mL/m^2^] SD 4.7	28 (19, 37)	27 (17, 36)	25 (16, 35)	24 (15, 33)	23 (13, 32)	21 (12, 31)
LAPEF [%] SD 6.4	47 (34, 59)	43 (31, 56)	40 (27, 52)	36 (23, 49)	32 (20, 45)	29 (16, 41)
LAPEI [%] SD 19.3	88 (51, 126)	79 (41, 117)	69 (31, 107)	59 (21, 97)	49 (11, 87)	40 (2, 77)
LAPPE [%] SD 8.3	75 (59, 91)	70 (54, 86)	65 (48, 81)	59 (43, 76)	54 (38, 70)	49 (33, 65)
LApPER [mL/s] SD 74.1	332 (187, 477)	289 (144, 435)	247 (101, 392)	204 (59, 349)	161 (16, 307)	119 (−27, 264)
LApPER/BSA [mL/s/m^2^] SD 37.9	194 (120, 269)	169 (95, 243)	144 (70, 218)	119 (44, 193)	94 (19, 168)	68 (−6, 143)
LAAEV [mL] SD 4.9	12 (2, 21)	13 (4, 23)	15 (5, 25)	17 (7, 26)	18 (9, 28)	20 (10, 30)
LAAEV/BSA [mL/m^2^] SD 2.6	7 (2, 12)	8 (3, 13)	9 (4, 14)	10 (5, 15)	11 (6, 16)	12 (6, 17)
LAAEI [%] SD 16.5	49 (17, 82)	52 (20, 85)	55 (23, 88)	58 (26, 91)	61 (29, 94)	64 (32, 97)
LAPAE [%] SD 8.3	26 (10, 42)	31 (14, 47)	35 (19, 51)	40 (23, 56)	44 (28, 61)	49 (33, 65)
LApAER [mL/s] SD 51.8	138 (37, 240)	154 (52, 255)	169 (68, 271)	185 (83, 286)	200 (99, 302)	216 (114, 317)
LApAER/BSA [mL/s/m2] SD 27.9	81 (27, 136)	90 (35, 145)	98 (44, 153)	107 (52, 162)	116 (61, 170)	124 (70, 179)
LAAEV/LAPEV [%] SD 21.8	31 (−12, 74)	45 (2, 87)	58 (15, 101)	72 (29, 115)	85 (42, 128)	99 (56, 142)

*LA* left atrium, *BSA* body surface area, *EI* expansion index, *TEF* total emptying fraction, *PEV* passive emptying volume, *CV* conduit volume, *PEF* passive emptying fraction, *PEI* passive emptying index, *pPER* peak passive emptying rate, *PPE* percentage of passive emptying, *AEV* active emptying volume, *AEI* active emptying index, *pAER* peak active emptying rate, *PAE* percentage of active emptying

**Table 4:** Left atrial function parameters significantly influenced by age in males (mean, 95 % confidence interval)

	20–29 years	30–39 years	40–49 years	50–59 years	60–69 years	70–79 years
LATEF [%] SD 5.3	61 (50, 71)	60 (49, 70)	58 (48, 69)	57 (47, 68)	56 (46, 67)	55 (45, 66)
LAEI [%] SD 31.6	156 (94, 218)	150 (88, 212)	145 (83, 207)	139 (77, 201)	133 (71, 195)	127 (65, 189)
LAPEV [mL] SD 6.5	33 (20, 46)	31 (18, 44)	28 (15, 41)	26 (13, 38)	23 (10, 36)	20 (7, 33)
LAPEV/BSA [mL/ m^2^] SD 3.2	17 (11, 24)	16 (9, 22)	14 (8, 21)	13 (7, 19)	12 (5, 18)	10 (4, 17)
LACV [mL] SD 10.3	61 (41, 81)	59 (39, 79)	57 (37, 78)	55 (35, 76)	53 (33, 74)	51 (31, 72)
LACV/BSA [mL/m^2^] SD 4.7	31 (22, 41)	30 (21, 40)	29 (20, 39)	28 (19, 37)	27 (18, 36)	26 (17, 35)
LAPEF [%] SD 5.6	42 (31, 53)	39 (28, 50)	35 (24, 46)	32 (21, 43)	29 (18, 40)	25 (14, 36)
LAPEI [%] SD 13.2	72 (46, 98)	64 (38, 90)	57 (31, 83)	49 (23, 75)	41 (15, 67)	34 (8, 60)
LAPPE [%] SD 7.1	70 (56, 84)	65 (51, 79)	60 (46, 74)	55 (41, 69)	50 (36, 64)	46 (32, 60)
LApPER [mL/s] SD 62.7	334 (211, 457)	296 (173, 419)	257 (134, 380)	219 (96, 342)	180 (57, 303)	142 (19, 265)
LApPER/BSA [mL/s/m^2^] SD 32.5	173 (109, 237)	153 (89, 216)	132 (68, 196)	112 (48, 175)	91 (28, 155)	71 (7, 135)
LAAEV [mL] SD 4.5	14 (5, 23)	16 (7, 25)	18 (9, 27)	20 (12, 29)	22 (14, 31)	24 (16, 33)
LAAEV/BSA [mL/m^2^] SD 2.2	7 (3, 12)	8 (4, 13)	9 (5, 14)	10 (6, 15)	11 (7, 16)	12 (8, 17)
LAAEF [%] SD 5.7	32 (21, 44)	34 (23, 45)	36 (24, 47)	37 (26, 48)	39 (28, 50)	40 (29, 52)
LAAEI [%] SD 13.8	49 (21, 76)	53 (26, 80)	57 (30, 84)	61 (34, 88)	65 (38, 92)	69 (42, 96)
LAPAE [%] SD 7.1	30 (16, 44)	35 (21, 49)	40 (26, 54)	45 (31, 59)	50 (36, 64)	54 (40, 68)
LApAER [mL/s] SD 57.4	183 (71, 296)	201 (88, 314)	219 (106, 331)	236 (124, 349)	254 (141, 366)	271 (159, 384)
LApAER/BSA [mL/s/m2] SD 29.1	96 (39, 153)	104 (47, 161)	112 (55, 169)	120 (63, 177)	128 (71, 185)	136 (79, 193)
LAAEV/LAPEV [%] SD 27.1	40 (−14, 93)	56 (3, 109)	72 (19, 126)	89 (35, 142)	105 (52, 158)	121 (68, 174)

*LA* left atrium, *TEV* total emptying volume, *BSA* body surface area, *EI* expansion index, *TEF* total emptying fraction, *PEV* passive emptying volume, *CV* conduit volume, *PEF* passive emptying fraction, *PEI* passive emptying index, *pPER* peak passive emptying rate, *PPE* percentage of passive emptying, *AEV* active emptying volume, *AEF* active emptying fraction, *AEI* active emptying index, *pAER* peak active emptying rate, *PAE* percentage of active emptying

BSA was significantly higher in males than in females (*p <* 0.001). On multivariate analysis, BSA was found to have significant independent effect on all the volumes (TEV, PEV, CV, and AEV) and on pPER and pAER. (*p <* 0.01 for all). A significant interaction between BSA and gender was seen, since in females BSA affected all the above mentioned parameters while in males it had only a significant effect on CV (*p <* 0.001).

### Effect of gender on LA function

Regarding parameters of reservoir function, absolute TEV was larger in males (*p =* 0.029) and absolute EI was larger in females (*p =* 0.04), but these differences disappear after normalization to BSA. With respect to parameters of conduit function, CV, PEF, PEI, PPE were larger in females (*p <* 0.01), but after normalization to BSA only CV was larger in males (*p <* 0.01) and PEV and PPE were larger in females (*p <* 0.01). For booster pump function, the majority of absolute parameters AEV, pAER, PAE were larger in males (all *p <* 0.05) but when normalized to BSA only AEV/PEV remained significantly larger in males. On multivariate analysis, gender had significant independent influence on EI, PEV, CV, PEF, PEI, PPE and PAE.

### Effect of age on LA function

Effect size of age on LA function is shown in Table 5. Absolute and normalized to BSA parameters of reservoir (except absolute and normalized TEV) and conduit function were lower, and booster pump function parameters (except AEF) higher in older female groups (all *p <* 0.01). Similar findings were obtained in males, except for absolute and normalized TEV and CV (*p <* 0.01). Accordingly AEV/PEV were higher in older groups (*p <* 0.001). On multivariable analysis age showed a significant (*p <* 0.01) influence on all absolute and normalized parameters except on absolute and normalized TEV, with lower values for reservoir and conduit function parameters and higher values of booster pump function parameters in older age groups. Age correlated with all absolute and normalized parameters of LA function except with absolute and normalized TEV. The strongest correlation was found with pPER, PPE and PAE, all *r =* 0.70.

**Table 5:** Effect size of age on left atrial function parameters for the whole group

	B	Std error	Beta	95 % CI
LATEF [%]	−0.13	0.03	−0.36	−0.2, −0.07
LAEI [%]	−0.8	0.2	−0.337	−1.2, −0.4
LAPEV [mL]	−0.26	0.03	−0.58	−0.32, −0.19
LAPEV/BSA [mL/m^2^]	−0.14	0.02	−0.59	−0.18, −0.11
LACV [mL]	−0.22	0.04	−0.31	−0.32, −0.13
LACV/BSA [mL/m^2^]	−0.12	0.03	−0.37	−0.17, −0.007
LAPEF [%]	−0.35	0.03	−0.68	−0.42, −0.28
LAPEI [%]	−0.87	0.09	−0.64	−1.05, −0.69
LAPPE [%]	−0.47	0.04	−0.697	−0.56, −0.39
LApPER [mL/s]	−4.21	0.36	−0.73	−4.93, −3.49
LApPER/BSA [mL/s/m^2^]	−2.29	0.20	−0.73	−2.68, −1.90
LAAEV [mL]	0.176	0.025	0.50	0.13, 0.23
LAAEV/BSA [mL/m^2^]	0.095	0.014	0.54	0.07, 0.12
LAAEF [%]	0.138	0.038	0.32	0.06, 0.21
LAAEI [%]	0.38	0.08	0.39	0.21, 0.54
LAPAE [%]	0.47	0.043	0.697	0.39, 0.56
LApAER [mL/s]	1.50	0.29	0.39	0.95, 2.11
LApAER/BSA [mL/s/m2]	0.82	0.16	0.43	0.51, .14
LAAEV/LAPEV [%]	1.5	0.14	0.70	1.22, 1.77

*LA* left atrium, *TEF* total emptying fraction, *EI* expansion index, *PEV* passive emptying volume, *BSA* body surface area, *CV* conduit volume, *PEF* passive emptying fraction, *PEI* passive emptying index, *PPE* percentage of passive emptying, *pPER* peak passive emptying rate, *AEV* active emptying volume, *AEF* active emptying fraction, *AEI* active emptying index, *PAE* percentage of active emptying, *pAER* peak active emptying rate

### Parameters of RA function

Results for the entire study group and male and female groups, without age breakdown, are shown in Table 6 and, for those variables with significant differences with age, results across age deciles are shown in Tables 7, 8 and 9. Figs. 2b, 3b and 4b show the main parameters plotted against age, normalized to BSA when appropriate.

**Table 6:** Right atrial function reference parameters summary data for all ages (mean, 95 % confidence interval)

	All	Males	Females
RATEV [mL] SD 11.9 *	56 (33, 79)	59 (36, 83)	53 (31, 75)
RATEV/BSA [mL/m^2^] SD 6.0	31 (19, 42)	30 (18, 43)	31 (20, 42)
RATEF [%] SD 7.0 * **	56 (42, 70)	54 (40, 68)	58 (46, 69)
RAEI [%] SD 40.1 * **	134 (55, 213)	123 (43, 202)	144 (67, 220)
RAPEV [mL] SD 9.5 *	36 (17, 54)	37 (15, 59)	36 (19, 52)
RAPEV/BSA [mL/ m^2^] SD 5.0	20 (10, 30)	19 (8, 30)	21 (12, 29)
RACV [mL] SD 12.9 **	40 (15, 66)	52 (22, 82)	28 (9, 47)
RACV/BSA [mL/m^2^] SD 7.2	19 (5, 33)	20 (5, 34)	17 (3, 31)
RAPEF [%] SD 7.3 * **	36 (22, 51)	34 (18, 50)	38 (26, 51)
RAPEI [%] SD 20.2 * **	62 (23, 102)	54 (14, 94)	66 (29, 103)
RAPPE [%] SD 9.7 * **	65 (46, 84)	63 (42, 84)	67 (50, 84)
RApPER [mL/s] SD 79 .4 *	245 (89, 401)	256 (115, 396)	225 (66, 403)
RAAEV [mL] SD 6.7 * **	19 (6, 32)	21 (7, 36)	13 (4, 23)
RAAEF [%] SD 8.3 *	30 (14, 46)	30 (12, 47)	30 (15, 45)
RAAEI [%] SD 17.1	45 (12, 78)	44 (7, 81)	46 (17, 75)
RApAER [mL/s] SD 78.6 *	219 (65, 373)	246 (86, 405)	191 (47, 336)
RAPAE [%] SD 9.1 * **	35 (17, 53)	37 (19, 56)	33 (16, 50)
RApAER/BSA [mL/s/m^2^] SD 42.3	118 (35, 201)	125 (45, 205)	111 (27, 196)
RAAEV/RAPEV [%] SD 28.2 *	61 (5, 116)	68 (2, 134)	53 (12, 95)

*RA* right atrium, *TEV* total emptying volume, *BSA* body surface area, *EI* expansion index, *TEF* total emptying fraction, *PEV* passive emptying volume, *CV* conduit volume, *PEF* passive emptying fraction, *PEI* passive emptying index, *pPER* peak passive emptying rate, *PPE* percentage of passive emptying, *AEV* active emptying volume, *AEF* active emptying fraction, *AEI* active emptying index, *pAER* peak active emptying rate, *PAE* percentage of active emptying, *SD* standard deviation for the whole group

* Significant differences (*p?<*?0.05) among age groups on multivariate analysis

** Significant differences (*p?<*?0.05) between males and females on multivariate analysis

**Table 7:** Right atrial function parameters significantly influenced by age in the whole group (mean, 95 % confidence interval)

	20–29 years	30–39 years	40–49 years	50–59 years	60–69 years	70–79 years
RATEV (mL) SD 11.9	62 (39, 85)	60 (37, 83)	58 (34, 81)	55 (32, 79)	53 (30, 76)	51 (27, 74)
RATEV/BSA (mL/m2) SD 6.0	34 (23, 46	33 (21, 45)	31 (20, 43)	30 (18, 42	29 (17, 40	27 (15, 39
RATEF [%] SD 7.0	60 (46, 74)	58 (45, 72)	57 (43, 70)	55 (41, 69)	54 (40, 67)	52 (38, 66)
RAEI [%] SD 40.2	161 (82, 240)	151 (72, 229)	140 (61, 219)	130 (51, 209)	120 (41, 198)	109 (31, 188)
RAPEV [mL] SD 9.5	45 (27, 64)	42 (23, 60)	38 (19, 56)	34 (15, 53)	30 (12, 49)	27 (8, 45)
RAPEV/BSA [mL/ m^2^] SD 5.0	25 (16, 35)	23 (14, 33)	21 (11, 31)	19 (9, 29)	17 (7, 27)	15 (5, 25)
RAPEF [%] SD 7.3	45 (31, 59)	42 (27, 56)	38 (24, 53)	35 (20, 49)	32 (18, 46)	29 (14, 43)
RAPEI [%] SD 20	84 (45, 127)	76 (37, 118)	67 (28, 109)	59 (20, 99)	51 (12, 90)	43 (3, 82)
RAPPE [%] SD 9.7	75 (56, 94)	71 (52, 90)	67 (48, 86)	63 (44, 82)	59 (40, 78)	55 (36, 74)
RApPER [mL/s] SD 79.4	351 (195, 506)	310 (154, 466)	269 (114, 425)	229 (73, 384)	188 (33, 344)	148 (-8, 303)
RAAEV [mL] SD 6.7	15 (2, 28)	17 (4, 30)	18 (5, 31)	20 (7, 33)	22 (8, 35)	23 (10, 36)
RAAEF [%] SD 8.3	2 (15, 41)	29 (16, 42)	30 (17, 43)	31 (17, 44)	31 (18, 45)	32 (19, 46)
RAPAE [%] SD 9.0	24 (7, 42)	29 (11, 46)	33 (15, 50)	37 (19, 55)	41 (23, 59)	45 (27, 63)
RApAER [mL/s] SD 78.6	19 (37, 245)	201 (47, 355)	212 (58, 366)	223 (69, 377)	234 (80, 388)	244 (90, 398)
RAAEV/RAPEV [%] SD 28.2	32 (-24, 87)	43 (-12, 98)	54 (-1, 109)	65 (10, 120)	76 (21, 132)	87 (32, 143)

*RA* right atrium, *TEV* total emptying volume, *BSA* body surface area, *EI* expansion index, TEF, total emptying fraction, *PEV* passive emptying volume, *CV* conduit volume, *PEF* passive emptying fraction, *PEI* passive emptying index, *pPER* peak passive emptying rate, *PPE* percentage of passive emptying, *AEV* active emptying volume, *AEF* active emptying fraction, *AEI* active emptying index, *pAER* peak active emptying rate, *PAE* percentage of active emptying

**Table 8:** Right atrial function parameters significantly influenced by age in females (mean, 95 % confidence interval)

	20–29 years	30–39 years	40–49 years	50–59 years	60–69 years	70–79 years
RATEV (mL) SD 11.1	61 (39, 83)	58 (36, 80)	55 (33, 77)	52 (30, 74)	49 (27, 71)	46 (24, 67)
RATEV/BSA (mL/m2) SD 5.6	36 (25, 47)	34 (23, 45)	32 (21, 43)	30 (19, 41)	28 (17, 39)	26 (15, 37)
RATEF [%] SD 5.8	63 (52, 75)	61 (50, 73)	59 (48, 71)	57 (46, 69)	55 (44, 67)	53 (42, 64)
RAEI [%] SD 40.8	177 (101, 254)	164 (88, 241)	151 (75, 228)	138 (62, 215)	125 (49, 202)	112 (36, 189)
RAPEV [mL] SD 8.4	46 (29, 62)	42 (25, 58)	38 (21, 55)	34 (17, 51)	30 (14, 47)	26 (10, 43)
RAPEV/BSA [mL/ m^2^] SD 5.7	24 (13, 35)	22 (11, 33)	20 (9, 31)	18 (7, 29)	16 (5, 27)	14 (3, 25)
RAPEF [%] SD 6.3	47 (35, 60)	44 (32, 56)	40 (28, 53)	37 (25, 49)	33 (21, 46)	30 (18, 42)
RAPEI [%] SD 19	90 (53, 127)	81 (43, 118)	71 (34, 109)	62 (25, 99)	53 (16, 90)	44 (6, 81)
RAPPE [%] SD 8.4	76 (59, 92)	72 (56, 89)	69 (52, 86)	66 (49, 82)	62 (46, 79)	59 (42, 75)
RApPER [mL/s] SD 85.9	352 (183, 520)	307 (138, 475)	262 (93, 430)	216 (48, 385)	171 (3, 340)	126 (-42, 295)
RAPAE [%] SD 8.4	24 (8, 41)	28 (11, 44)	31 (14, 48)	34 (18, 51)	38 (21, 54)	41 (25, 58)
RAAEV/RAPEV [%] SD 21.1	30 (-12, 71)	39 (-3, 80)	48 (6, 899	57 (15, 98)	66 (24, 107)	74 (33, 116)

*RA* right atrium, *TEV* total emptying volume, *BSA* body surface area, *EI* expansion index, *TEF* total emptying fraction, *PEV* passive emptying volume, *CV* conduit volume, *PEF* passive emptying fraction, *PEI* passive emptying index, *pPER* peak passive emptying rate, *PPE* percentage of passive emptying, *AEV* active emptying volume, *AEF* active emptying fraction, *AEI* active emptying index, *pAER* peak active emptying rate, *PAE* percentage of active emptying

**Table 9:** Right atrial function parameters significantly influenced by age in males (mean, 95 % confidence interval)

	20–29 years	30–39 years	40–49 years	50–59 years	60–69 years	70–79 years
RATEV/BSA (mL/m2) SD 6.4	33 (20, 46)	32 (19, 45)	31 (18, 43)	30 (17, 42)	29 (16, 41)	28 (15, 40)
RAEI [%] SD 40.8	139 (59, 219)	133 (53, 213)	126 (46, 206)	120 (40, 200)	113 (33, 193)	107 (27, 187)
RAPEV [mL] SD 11.4	47 (24, 69)	43 (20, 65)	39 (17, 62)	36 (13, 58)	32 (9, 54)	28 (6, 51)
RAPEV/BSA [mL/ m^2^] SD 5.7	24 (13, 35)	22 (11, 33)	20 (9, 31)	18 (7, 29)	16 (5, 27)	14 (3, 25)
RAPEF [%] SD 8.2	42 (26, 58)	39 (23, 55)	36 (20, 52)	33 816, 49)	29 (13, 46)	26 (10, 42)
RAPEI [%] SD 20.3	73 (33, 113)	66 (26, 106)	59 (19, 98)	51 (11, 91)	44 (4, 84)	37 (-3, 76)
RAPPE [%] SD 10.8	75 (54, 96)	70 (49, 92)	66 (44, 87)	61 (40, 82)	57 (35, 78)	52 (31, 73)
RApPER [mL/s] SD 71.6	351 (210, 491)	314 (174, 454)	278 (137, 418)	241 (101, 381)	204 (64, 345)	168 (28, 308)
RAAEV [mL] SD 7.3	15 (1, 30)	18 (3, 32)	20 (6, 34)	22 (8, 37)	25 (10, 39)	27 (13, 41)
RAAEF [%] SD 8.2	42 (26, 58)	39 (23, 55)	36 (20, 52)	33 816, 49)	29 (13, 46)	26 (10, 42)
RApAER [mL/s] SD 81.3	208 (49, 367)	223 (63, 382)	237 (78, 397)	252 (92, 411)	266 (107, 426)	281 (121, 440)
RAPAE [%] SD 9.5	25 (6, 44)	30 (11, 48)	34 (16, 53)	39 (20, 58)	44 (25, 63)	49 (30, 67)
RApAER/BSA [mL/s/m2] SD 40.8	109 (29, 189)	115 (35, 195)	122 (42, 202)	128 (48, 208)	134 (54, 214)	141 (61, 221)
RAAEV/RAPEV [%] SD 33.6	34 (-32, 100)	47 (-19, 113)	60 (-6, 126)	73 (7, 139)	87 (21, 153)	100 (34, 166)

*RA* right atrium, *TEV* total emptying volume, *BSA* body surface area, *EI* expansion index, *TEF* total emptying fraction, *PEV* passive emptying volume, *CV* conduit volume, *PEF* passive emptying fraction, *PEI* passive emptying index, *pPER* peak passive emptying rate, *PPE* percentage of passive emptying, *AEV* active emptying volume, *AEF* active emptying fraction, *AEI* active emptying index, *pAER* peak active emptying rate, *PAE*, percentage of active emptying

On multivariate analysis, BSA was found to have significant independent influence on TEV, PEV, CV and pAER.

### Effect of gender on RA function

Regarding parameters of reservoir function, TEV was larger in males (*p =* 0.029) while EI and TEF were both larger in females (*p =* 0.005 and *p =* 0.001, respectively). The majority of parameters of conduit function were larger in females (PEF, PEI, PPE, all *p <* 0.01), except for CV that was larger in males (*p <* 0.001). For booster pump function, the majority of parameters (AEV, pAER, PAE) as well as AEV/PEV were larger in males (all *p <* 0.05). On multivariate analysis, gender had significant independent influence on absolute EI, TEF, PEF, PEI, PPE, AEV, PAE, normalized PEV and pPER and absolute and normalized CV.

### Effect of age on RA function

Effect size of age on RA function is shown in Table 10. In females there was a significant decrease in reservoir function parameters with increasing age (all *p <* 0.001), a decrease in the majority of conduit function parameters, except absolute and normalized CV, and an increase in PAE and AEV/PEV (all *p <* 0.05). In males age did not influence reservoir function parameters, while there was a significant decrease in all conduit function parameters, except absolute and normalized CV, and an increase in all booster pump function parameters except AEI (all *p <* 0.05). On multivariate analysis, age was independent predictor of all absolute and normalized parameters of reservoir function (all *p <* 0.001), on all absolute and normalized parameters of conduit function except absolute and normalized CV (all *p <* 0.05), and on all booster pump function parameters except AEI (*p <* 0.05). Age correlated with all absolute and normalized parameters of RA function except with normalized TEV, absolute and normalized CV and with AEI. The strongest correlation was found with AEV/PEV (*r =* 0.70).

**Table 10:** Effect size of age on right atrial function parameters for the whole group

	B	Std error	Beta	95 % CI
RATEV [mL]	−0.24	0.067	−0.306	−0.38, −0.11
RATEV/BSA [mL/ m^2^]	−0.13	0.04	−0.33	−0.2, −0.06
RATEF [%]	−0.157	0.039	−0.337	−0.23, −0.08
RAEI [%]	−1.06	0.22	−0.39	−1.51, −0.62
RAPEV [mL]	−0.40	0.05	−0.57	−0.51, −0.30
RAPEV/BSA [mL/ m^2^]	−0.21	0.03	−0.58	−0.27, −0.16
RAPEF [%]	−0.33	0.04	−0.58	−0.41, −0.25
RAPEI [%]	−0.89	0.11	−0.585	−1.109, −0.67
RAPPE [%]	−0.41	0.05	−0.57	−0.52, −0.31
RApPER [mL/s]	−4.17	0.44	−0.67	−5.03, −3.30
RAAEV [mL]	0.15	0.04	0.35	0.08, 0.23
RAAEF [%]	0.10	0.04	0.20	0.01, 0.19
RAPAE [%]	0.42	0.05	0.60	0.32, 0.52
RApAER [mL/s]	0.96	0.43	0.19	0.11, 1.81
RAAEV/LAPEV [%]	1.11	0.16	0.54	0.80, 1.42

*RA* right atrium, *TEV* total emptying volume, *BSA* body surface area, *TEF* total emptying fraction, *EI* expansion index, *PEV* passive emptying volume, *PEF* passive emptying fraction, *PEI* passive emptying index, *PPE* percentage of passive emptying, *pPER* peak passive emptying rate, *AEV* active emptying volume, *AEF* active emptying fraction, *PAE* percentage of active emptying, *pAER* peak active emptying rate

## Discussion

This study using state-of-the-art CMR acquisition techniques and volumetric analysis provides a reference for normality for all parameters of left and right atrial function, adjusted for the effect of age and gender. Despite considerable data demonstrating the utility of atrial function in predicting risk in several heart conditions, strategies incorporating these parameters have not been exploited in clinical practice, due to several reasons among them the paucity of normative values. These data, then, have significant clinical and research utility, and both tables and graphical display are included for clinical use.

### Left atrial function

As a continuum of the left ventricle (LV), especially during diastole, LA size and function are very much influenced by ventricular compliance. LA size is a powerful predictor of adverse cardiovascular outcomes, but LA function has not been so extensively elaborated [20]. Technical advances have allowed the non-invasive characterization and quantitation of LA function with imaging techniques, including echocardiography, cardiac computed tomography [21], nuclear scintigraphy and CMR. Echocardiography is the simplest and most cost-effective method and has been validated for the study of LA function with two-dimensional linear and volumetric measurements [22], pulsed wave Doppler [23], acoustic quantification [24], tissue Doppler [25] and speckle tracking imaging [26]. Still, problems with acoustic window and reproducibility may affect their use. As for cardiac computed tomography and nuclear scintigraphy, the low temporal resolution and need for contrast and radiopharmaceutical agents limit their use.

CMR provides very accurate and reproducible volumetric measurements of both atria with the short axis method along the cardiac cycle, and it is the gold standard technique for the assessment of atrial volumes [16, 17]. The area-length method is more frequently used in clinical practice since it does not require additional acquisitions and the analysis is faster, but it relies on geometric assumptions and is less reproducible. More recently, CMR feature tracking analysis has been introduced that provides a faster assessment of LA function, with a loss of reproducibility as it also relies on the area length method [27, 28]. In our study we have obtained with the short axis method a reference range for all parameters of LA reservoir, conduit and booster pump functions, with differentiation into all subjects, males and females, and sub-division into age groups and absolute and BSA normalized values. For the whole group we obtained a value for total LA emptying—or ejection—fraction of 59 ± 5.8 %, 35 ± 6 % for passive emptying fraction and 36 ± 6.8 % for active emptying fraction. Interestingly, the confidence intervals are wide and this could be due to the variable shape of the atria in normal subjects, resulting in a wide range of normal values in both men and women. Also, there are some values, both for LA and RA, where a negative confidence interval value is observed. This is only a result of the statistical modeling but should not be considered clinically as it is not physiologically possible. Very few data obtained with CMR are available to compare our results. Raman [29] found in a small group of 15 controls studied with either SSFP or gradient echo sequences a TEF of 32 ± 5 %, hardly comparable to our results due to methodological differences. Hudsmith [30], using the biplane area-length method in 108 healthy subjects, obtained a TEF of 54 ± 12 %. Le Ven [31], using a short axis volumetric method in 434 healthy adults, found LA ejection fraction of 59 ± 8 % for males and 61 ± 7 % for females. We have not found reports on other parameters measured with CMR for comparison. These have been measured with different echocardiographic techniques but they are not comparable [22, 24].

We observed that atrial volumes were associated to BSA, which were then normalized to this parameter. On multivariate analysis gender affected only one reservoir function parameter, EI, and one booster pump function marker, PAE, while it had a significant independent influence on most conduit function parameters including PEV, CV, PEF, PEI and PPE. There are very few CMR studies with which to compare our results. Le Ven [31] observed that gender was independently associated with LA ejection fraction (equivalent to TEF in our study) while Hudsmith [30] reported no differences in LA ejection fraction between males and females. Data are then controversial and in none of those studies were specific parameters of conduit or booster pump function measured. More data are available from echocardiography. Nikitin [32], with 2D echocardiography in 123 healthy volunteers, showed no differences with gender in LA function parameters, including EI, PEF and AEF. Accordingly, Morris [33] in 329 healthy adults studied with speckle tracking echocardiography showed no differences in peak atrial strain rate during atrial contraction and peak atrial strain during atrial relaxation, which would be concordant with our findings. Interestingly, though there is no clear knowledge or explanation for gender differences in atrial function, an animal study [34] has shown sexually dimorphic responses to extracellular calcium, isoproterenol and phenylephrine which would suggest a possible role of sex hormones in these differences.

We found differences between younger and older individuals for all absolute and normalized parameters, except on absolute and normalized TEV, with significantly lower reservoir and conduit function parameters and higher booster pump function parameters in the older age groups. There is a general agreement, using different imaging techniques, over the significant effect of age on global and regional LA function [22, 24, 35]. The decline in passive emptying probably represents an age related change in left ventricular properties leading to diastolic dysfunction, with an increase in active emptying which compensates for the decrease in early diastolic filling. Nevertheless, Hudsmith [30] found no influence of age on LA ejection fraction and Le Ven [31] did not assess this. The majority of studies have been carried out with varied echocardiographic techniques and results support our findings notwithstanding the methodological differences. Nikitin [32] found no effect of age on reservoir function, while there was a progressive decrease in conduit function and an increase in booster pump function with age. Triposkiadis [22] with pulsed wave Doppler echocardiography observed a clear effect of age on LA function with findings very similar to our study. Okamatsu [36], with two-dimensional speckle tracking echocardiography in 140 volunteers also observed that aging significantly decreases LA conduit function and increases booster function.

The differences observed with age might have clinical importance. For instance, in 1802 participants in the Dallas Heart Study [37] it was shown the incremental prognostic value of LA ejection fraction (LAEF), measured with the area-length method, beyond traditional risk factors, LV ejection fraction, and LV mass: decreasing LAEF [hazard ratio per 1 standard deviation, 8.0 %) was independently associated with mortality. In this study a significant association of LAEF with age was seen. If we examine that single parameter of LA function in our study, the lower limit of normality for LATEF for the whole group, as shown in Table 2, was 51 % in the younger age group and 44 % in the older age group which represents a decrease of 13 %, or 1.2 standard deviations. Consequently, even just for this single parameter the differences seen with age are sufficiently large and they would affect the clinical interpretation of the results. In another investigation carried out with echocardiographic techniques, LA EF was shown to be a powerful independent predictor of new-onset atrial fibrillation and atrial flutter in 574 elderly participants [20]. Patients at highest risk were those with both LAEF <49 % and LAVi >38 ml/m2, and LAEF was superior and incremental to LAV. In our CMR study, though we are aware that there is not an equivalence between parameters measured with echocardiography and CMR, a LATEF of 49 % would be abnormal in young people but it could be normal in the older.

### Right atrial function

RA function assessment may have an important clinical impact for the management of patients with right heart disease. CMR offers excellent visualization of the right heart and is the technique of choice for the quantification of right atrial and ventricular volumes, though RA volume and function measurements with the short axis method are not routinely performed. The area-length method is faster but again problems with accuracy and reproducibility limit its use. In our study we have obtained with the short axis method a reference range for all parameters of RA reservoir, conduit and booster pump functions, with differentiation into all subjects, males and females, and sub-division into age groups and absolute and BSA-normalized values when applicable. For the whole group we obtained a value for TEF of 56 ± 7.0 %, 36 ± 7.3 % for PEF and 30 ± 8.3 % for AEF. Previous works have mostly been done with different subject selection, sequence, acquisition protocol or analysis method, so they are hardly comparable [38]. Raman et al. [29] found a TEF of 31 ± 9 % in a small group of young volunteers studied with a different protocol and variable acquisition sequence. Sievers et al. [39] measured RA ejection fraction in a group of 70 healthy subjects with SSFP cines acquired both with the area-length and the short axis methods and obtained a reference value for RA ejection fraction of 47.2 ± 8.3 %, which is slightly lower than ours. Differences in the age range of the subjects included might account for the differences. Though echocardiography has been most widely used, values are hardly comparable Willens et al. [40] studied with echocardiography 57 healthy subjects (30 subjects < 60 year and 27 subjects ≥ 60 years of age) and observed in those ≥ 60 years a PEF and AEF of 46 ± 23 % and 54 ± 23 %, and in the subjects under 60 year a PEF and AEF of 60 ± 15 % and 40 ± 15 %, all of these values are higher than ours but the different imaging technique and methodology used are a major obstacle for comparison. 3D-echocardiography has been claimed to be comparable to CMR for ventricular measurements. Peluso et al. [41] studied 200 healthy subjects aged 18–75 years and found with 3D-echocardiography an overall TEF of 63 ± 9 %, PEF of 46 ± 11 % and AEF of 31 ± 8 %, slightly higher than ours. Aune et al. [42] found in 166 subjects also studied with 3D-echocardiography a reference value of 46 % for TEF, which was lower than our values.

On multivariate analysis gender had significant independent influence on most parameters of reservoir (EI, TEF), conduit (PEF, PEI, PPE, pPER, absolute and normalized CV, normalized PEV) and booster pump functions (AEV, PAE). Sievers [39] found higher TEF values for TEF in females but differences were not significant. With echocardiography Peluso [41] found that TEF, PEF and AEF were all significantly higher in women, and found analogous results when speckle tracking echocardiography was used. Aune et al. [42] also found a higher TEF in females.

With respect to the effect of age, Sievers [39] found no relation of right atrial ejection fraction with age. On the contrary, some echocardiographic studies have shown results consistent with ours. Willens et al. [40] observed a clear effect of age on right atrial function. Peluso [41] also found a significant effect of age, with decrease of TEF and PEF, and increase of AEF with aging. We might hypothesize that with increasing age not only left ventricular diastolic function is affected, but also right ventricular diastolic function is compromised. These findings might have clinical impact, since in our population with a growing number of elderly people and age-related diseases such as atrial fibrillation, pulmonary hypertension and RV dysfunction, right atrial function might need to be determined more frequently.

## Limitations

Time-volume curves have been obtained using a vendor independent software analysis, which might hypothetically have implications for the applicability of these reference values when other softwares are used. However, we used this software for obtaining reference values for atrial and ventricular dimensions and function and applicability of these data is now generalized.

Though our sample size was higher than in several studies, with equal distribution of males and females across all age groups, we did not recruit subjects over 80 years of age. However, the enrolment of truly healthy subjects over that age is difficult.

Finally, despite all subjects were asymptomatic with normal physical examination, normal electrocardiogram, normal BNP levels, no wall motion abnormalities, and no cardiovascular risk factor, we cannot exclude the possibility of mild subclinical disease particularly in older subjects.

## Conclusions

A significant difference in both LA and RA function is observed among young and old age groups. This consists mainly of lower passive components and higher active emptying in older individuals. Also, gender affects a number of, mainly, components of conduit and booster pump function. Thus the reference values provided are of significant clinical and research utility for the interpretation of CMR studies.

## Abbreviations

AEF: Active emptying (or ejection) fraction
AEI: Active emptying index
AEV: Active emptying volume
BSA: Body surface area
CMR: Cardiovascular magnetic resonance (CMR) provides high quality images of SSFP, steady state free precession cine sequences
CV: Conduit volume
EI: Total emptying index
LA: Left atrium
LV: Left ventricle
PAE: Percentage of active emptying
pAER: Peak active emptying rate
PEF: Passive emptying (or ejection) fraction
PEI: Passive emptying index
PEV: Passive emptying volume
PPE: Percentage of passive emptying
pPER: peak passive emptying rate
RA: Right atrium
TEF: Total emptying (or ejection) fraction
TEV: Total emptying volume
